# RISE-EM: Resident Instruction in Social Emergency Medicine, a Cohort Study of a Novel Curriculum

**DOI:** 10.5811/westjem.18103

**Published:** 2024-06-11

**Authors:** Heidi Roche, Brandon A. Knettel, Christine Knettel, Timothy Fallon, Jessica Dunn

**Affiliations:** * Department of Emergency Medicine, Maine Medical Center, Portland, Maine; † Tufts University School of Medicine, Portland, Maine; ‡ Duke University School of Nursing, Durham, North Carolina; § Duke Global Health Institute, Durham, North Carolina; ∥ Department of Emergency Medicine, University of North Carolina School of Medicine, Rex Hospital, Chapel Hill, North Carolina; ¶ Department of Emergency Medicine, Emory University School of Medicine, Atlanta, Georgia

## Abstract

There is recognition in the field of emergency medicine (EM) that social determinants of health (SDoH) are key drivers of patient care outcomes. Leaders in EM are calling for curricula integrating SDoH assessment and intervention, public health, and multidisciplinary approaches to EM care throughout medical school and residency. This intersection of SDoH and the emergency care system is known as social emergency medicine (SEM). Currently, there are few resources available for EM training programs to integrate this content; as a result, few EM trainees receive adequate education in SEM. To address this gap, we developed a four-part training in SEM tailored to EM residency programs and medical schools.

This curriculum, known as RISE-EM (Resident Instruction in Social Emergency Medicine), uses video lectures, case examples, and group discussions to engage trainees and develop competency in providing sound care that is grounded in evidence-based principles of SEM. In the current study, we tested RISE-EM by delivering the video lectures to residents and medical students in two training programs. We administered pre- and post-course knowledge tests and a post-course participant attitudes survey to assess the acceptability and potential efficacy of the program for improving SEM knowledge and attitudes among EM learners.

We found it to be both feasible and acceptable to introduce SEM content in residency conferences, with preliminary data showing statistically significant improvement in knowledge of the content and self-efficacy to apply it to their clinical practice. In summary, RISE-EM has been highly valued by EM learners and viewed as a strong supplement to their existing training, and it has been shown to successfully improve SEM knowledge and attitudes.

Population Health Research Capsule What do we already know about this issue?

*The intersection of social determinants of health and emergency medicine is an important area of training for which little open access training material exists.*

What was the research question?

*Can a social emergency medicine (SEM) curriculum increase resident learners’ SEM knowledge and self-efficacy?*

What was the major finding of the study?

*Our curriculum improved SEM knowledge and self-efficacy in a cohort of 26 students (P < 0.001).*

How does this improve population health?

*Open access education material for SEM can assist in facilitating the development of SEM skills and self-efficacy for residents in their clinical practice.*

## BACKGROUND

Health is closely intertwined with multiple complex aspects of a person’s daily life, an interaction termed social medicine. Several studies have demonstrated that social determinants of health (SDoH), which may include personal, social, economic, and other aspects of well-being, may contribute to 40% or greater of total health outcomes, whereas clinical interventions, both inpatient and outpatient, were estimated to contribute a mere 12–20%.^1^ For example, although the clinician may diagnose pneumonia and prescribe antibiotics, the pneumonia will not improve if the patient cannot access the treatment due to cost or other barriers or continues to live in an environment that does not allow or promote healing.^2^

In the 19^th^ century, Virchow stated: “Medicine is a social science, and politics is nothing more than medicine on a large scale.”^3^ However, only recently has the field of emergency medicine (EM) begun to appropriately emphasize the need for interventions beyond medical care, at both political and societal levels.^4^^,^^5^ Social medicine, a term that includes considerations of SDoH, social epidemiology, and social science in the provision of medical care, emphasizes concepts of health equity, advocacy and interdisciplinary approaches to improving patient outcomes and reducing health disparities.^6^

Given the large impact of social determinants on health, it seems natural to emphasize training in social medicine across the stages of medical education. Some undergraduate programs and medical schools have begun implementing new social medicine curricula; however, these modules continue to make up only a small segment of most training programs.^7^^–^^9^ In response to a growing body of research and interest in social medicine, medical leaders, including the Accreditation Council for Graduate Medical Education and the American College of Emergency PhysiciansACGME and ACEP, are calling for more exposure to social medicine throughout medical school and residency training.^10^^–^^12^

Many EM leaders have expressed valid concerns regarding the challenges of addressing SDoH in the ED, often based on lack of resources to effectively implement new services in an already overburdened system. The emergency department (ED) is perceived by many members of the community as a setting where they can seek support for unmet social needs, a pattern that places a substantial burden on care systems not designed for this purpose.^13^^,^^14^ However, as a system that provides care at all times, regardless of complaint or patient circumstance, the ED is arguably the care setting most critical for integrating principles of social medicine.^15^^–^^17^

This reevaluation of the role of EM has occurred in a changing climate of social welfare, where the ED has become part of a critical social safety net.^15^ It is becoming clear that it is no longer acceptable to treat the medical etiologies of health problems alone, when SDoH play such a key role in our patients’ experience of disease and illness. Given their frontline interaction with SDoH, emergency physicians are in a key position to lead a paradigm shift from merely treating downstream disease to leading change, systemically and collaboratively, in upstream preventative health factors.^4^^,^^15^^,^^17^^,^^18^ This intersection of SDoH and the emergency care system is known as social emergency medicine (SEM), a promising approach to responding to the unmet societal demands flooding the ED. Emergency clinicians must embrace an expanded role to guide the healthcare system and policymakers in designing a system that integrates social and medical aspects of care.^15^

Despite these escalating roles and responsibilities of the emergency care system, there has been little inclusion of social medicine in graduate EM education, and many EM education leaders have identified this as an area of need.^13^^,^^17^^–^^19^At the time this project was started, there were only four social medicine and population health fellowships in EM nationally. This number has grown to 11 by time of publication, reflecting the growing acknowledgment of this field.^20^ These residency tracks and fellowships are important in paving the way for the budding field of SEM but are harder to translate to other programs seeking to adopt SEM content.

One way to offer a curriculum or content that is easily adaptable into various programs is Free Open Access Medical Education (FOAMed). This open access education is prominent in EM, and existing online material focuses heavily on standard board exam content, procedural competence, and cutting-edge therapies. Given the paucity of SEM tools available online, projects are currently in the works to offer supplemental blog posts or cases covering SEM material. However, at this time, to the best of our knowledge, a unifying curriculum with objectives, ordered lectures, and supplemental material does not exist in FOAMed form, accessible to the greater EM education community. To address this gap in training resources for EM residents and medical students, we developed a four-part SEM training curriculum to be delivered by video with accompanying case examples and group discussions, known as Resident Instruction in Social Emergency Medicine (RISE-EM).

## OBJECTIVES

We describe the design of RISE-EM and findings from piloting the curriculum with three cohorts of EM residents and medical students. Our objective with these pilot cohorts was to test the preliminary feasibility, acceptability, and potential for impact of RISE-EM in facilitating the development of SEM skills for learners in their clinical practice. The three course objectives are as follows:

1. Expose EM residents and medical students to the concepts of SEM
2. Provide learners with a vocabulary that they can use to proactively address SDoH
3. Teach SEM skills that learners can use in the ED when working with patients

## CURRICULAR DESIGN

The RISE-EM curriculum was built upon a core foundation in social medicine principles and curriculum objectives from the Social Medicine Reference Toolkit.^6^ The toolkit was validated through an analytical review by 15 social medicine programs worldwide and published by a national organization of physicians and public health scientists known as the Social Medicine Consortium.

The SEM-specific material was developed using diverse published works, including a series from the Inventing Social Emergency Medicine Consensus Conference in 2017, a summit composed of leaders from many organizations, including the Andrew Levitt Center for Social Emergency Medicine and ACEP.^21^ We also reviewed the primary literature to identify challenges and successful techniques related to teaching SDoH content. Throughout the modules, difficult concepts were repeated, explained in multiple different ways, and incorporated into clinical scenarios to encourage understanding and depth of processing. “Nudges,” a theme throughout RISE-EM, were inspired by nudge theory, a concept in behavioral economics and political theory.^22^

**Figure 1.**
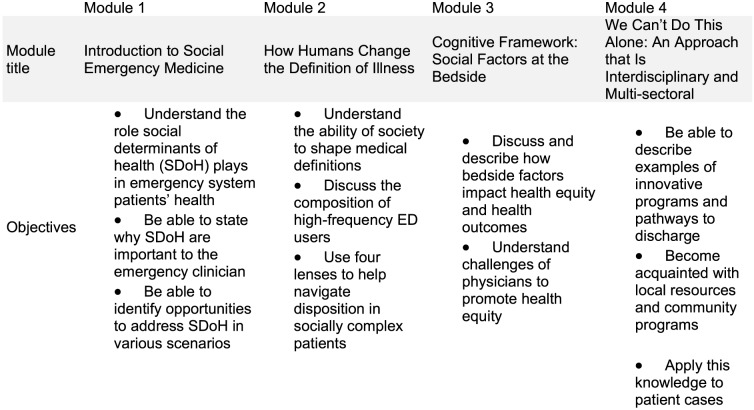
Course modules by individual objectives.

The RISE-EM curriculum is based on video lectures, which allows it to be used asynchronously or synchronously, in one sitting or over multiple sessions. The curriculum consists of four video modules (Figure 1), each approximately 20 minutes in length (Appendix A). The sessions were designed to be short enough to fit into most conferences or to hold the attention of a busy resident outside the hospital. The videos use motifs and engaging discussions carried through each video to encourage depth of processing and to assist with recall. The educational modality was chosen to facilitate easy adoption by EM residency and medical student training programs with teaching guides provided and the ability to fit into various didactic schedules and both in-person and virtual formats.

## METHODS

We completed a prospective cohort study designed to test the feasibility, acceptability, and potential efficacy of RISE-EM in improving SEM knowledge and attitudes among EM learners. We tested the curriculum with two groups of EM residents. Group 1 consisted of residents and medical students at a southeastern EM residency conference in October 2020. Group 2 consisted of residents at a northeastern EM residency conference in November 2021. We arranged for participation by sending an introductory email through each residency’s email listserv (Appendix B). Participants were given two weeks to complete pre-course material and two weeks to submit post-course material after the intervention. As this was a pilot feasibility study of an educational innovation, the study size was determined by the number of residents and medical students who chose to participate at the two institutions where the curriculum was tested. This research was conducted with the approval of each institution’s institutional review board (IRB).

Learners who wished to participate in the study followed a link in the introductory email, provided their informed consent to participate, and were asked to take a 20-minute pre-test and survey on a secure survey website, with responses collected anonymously. Study participants then watched the four video lectures (delivered differently to Group 1 and Group 2, see below). After watching all the videos, participants engaged in a live group discussion during standard residency didactic time. They were then asked to complete a second 20-minute online survey, comprising the same knowledge test and additional questions about the feasibility and acceptability of the course for future delivery. Reminder emails for completion were automatically sent to individuals every five days after initial pre-course material completion, for a maximum of up to three times as defined in our IRB application. This study protocol is illustrated in Appendix C.

### Survey Instruments

The pre-course survey began with basic demographic questions and eight additional questions related to interest and self-efficacy in applying SEM principles in clinical practice (Appendix D). The 19-item pre- and post- knowledge tests were identical, composed of 4–5 multiple-choice questions of content from each RISE-EM lecture, with 19 in total (Appendix E). Each correct response received 1 point for a total score of 0–19. The course content questions were designed to assess baseline and post-course SEM knowledge.

The post-course survey consisted of the same eight items to assess for change in interest and self-efficacy (ie, “Following my completion of this course, I feel confident in assessing and addressing social determinants of health in my clinical encounters”). Feasibility was assessed by recording the number of modules completed by each participant. We also evaluated acceptability and perceptions of course quality with nine questions adapted from the Student Evaluation of Educational Quality (SEEQ) instrument.^23^ The post-course survey concluded with open-ended questions regarding 1) specific recommendations for improving the course, 2) content that was most useful, 3) missing content or areas to add, and 4) ways the course changed their perspective on social medicine, if at all (see Appendix F for the full items).

### Statistical Analysis

We used descriptive statistics to summarize characteristics of the study sample. We assessed the potential efficacy of the RISE-EM curriculum by comparing participants’ pre-and post-curricular scores on knowledge and self-efficacy items using paired samples *t*-tests. Adequate feasibility was defined by a target of at least 80% of participants completing all four course modules and the post-course survey. For the acceptability and course quality questions derived from the SEEQ, we defined adequate acceptability as at least 80% of participants indicating that they fully agreed with the item. It is important to note that these quantitative comparisons are exploratory in nature due to the small sample size in this pilot study.

To analyze participant responses to open-ended questions, we used an applied thematic approach to qualitative analysis.^24^ Two study investigators read the responses independently to identify common themes and develop a preliminary codebook. The investigators then came together to discuss these preliminary themes, identify similarities and differences in the codebooks, and combine the themes into a single, cohesive codebook. The team then re-analyzed the qualitative responses onto the final codebook, defined the codes in descriptive memos, and reviewed the codes to identify representative quotations. We randomly selected five participants’ (26.3%) responses to be re-coded by a second reviewer and evaluated for inter-coder agreement using a pre-established threshold of 80% agreement.^25^ Inter-coder agreement on these responses was 90.5%, which exceeded the desired threshold, and disagreements identified in the re-coding process were reconciled by the two reviewers until consensus was reached.

## IMPACT/EFFICACY

### Participants

Participants in Group 1 watched the modules in conference over the course of an hour, and then engaged in a 20-minute group discussion. In total, six participants (of 36 total eligible trainees) in Group 1 enrolled in the study. All six enrolled participants completed both the pre- and post-test and survey material. In Group 2, 23 participants (of 30 total eligible trainees) enrolled in the study. Participants watched the video modules on their own and then had a 50-minute group discussion in conference. Two participants in Group 2 (8.3%) did not complete post-course material.

Although both groups had material presented during regularly scheduled educational sessions, Group 2 completed all video modules asynchronously immediately following the pre-course material, possibly explaining the higher rate of participation.

Participants had a mean age of 30 years and a relatively equal gender distribution (Table 1). The majority of participants who identified as White ethnicity (24, 83%) and a relatively even spread between levels of training at about one-third of participants per postgraduate yearPGY year in the combined cohort, plus two fourth-year medical students participating in Group 1. Baseline enthusiasm and interest was very high for SEM. Approximately half of participants reported prior coursework in social medicine, ranging from self-study, single lectures and discussions, or short workshops, to formal courses as a core component of the medical school curriculum.

**Table 1.** Participant demographics and other characteristics.

	Group 1 (n = 6) number (%)	Group 2 (n = 23) number (%)	Combined cohort (N = 29) number (%)
Age (years), mean (range)	29 (27–33)	30 (27–37)	30 (27–37)
Female gender	1 (17%)	13 (57%)	14 (48%)
Ethnicity			
White	4 (67%)	20 (87.0%)	24 (83%)
Black	1 (17%)	0 (0%)	1 (3%)
Hispanic or Latino	1 (17%)	0 (0%)	1 (3%)
Asian	0 (0%)	1 (4.3%)	1 (3%)
More than one race/ethnicity	0 (0%)	1 (4.3%)	1 (3%)
Declined to respond	0 (0%)	1 (4.3%)	1 (3%)
Level of training^1^			
MS4	2 (33%)	0 (0%)	2 (7%)
PGY-1	1(17%)	8 (34.8%)	9 (31%)
PGY-2	1 (17%)	9 (39.1%)	10 (35%)
PGY-3	2 (33%)	6 (26.1%)	8 (28%)
Considers SEM important			
“Yes”	6 (100%)	22 (96%)	28 (97%)
“Somewhat”	0 (0%)	1 (4%)	1 (3%)
Interested in learning more about SEM			
“Yes”	6 (100%)	19 (82.6%)	25 (86%)
“Somewhat”	0 (0%)	4 (17%)	4 (14%)
Prior coursework in social medicine	1 (17%)	15 (65.2%)	16 (55%)

*MS4*, fourth-year medical student; *PGY*, postgraduate year; *SEM*, social emergency medicine.

### Improvement in SEM Knowledge and Self-Efficacy

In Group 1, six participants completed pre-and post-course assessments. SEM knowledge significantly improved by 3.2 points on average, from 7.0 to 10.2 (*t*(5) = 3.63, *P* = 0.015), while self-efficacy significantly improved by 4.8 points on average, from 12.3 to 17.1 of 18 possible (*t*(5) = 3.24, *P* = 0.023). In Group 2, pre- and post-course assessments of the 21 participants also showed statistically significant improvement in both knowledge and self-efficacy (Table 2). Knowledge of SEM improved by 2.5 points on average, from 8.2 to 10.7 (*t*(20) = 4.07, *P* = 0.001). Self-efficacy also significantly improved by 5.8 points on average, from 8.0 to 13.8 (*t*(20) = 8.89, *P* < 0.001).

**Table 2.** Post-course test analysis showing change in \knowledge of social emergency medicine and self-efficacy and completion of modules (n = 27).

	Group 1 (n = 6)	Group 2 (n = 21)	Combined (n = 27)^1^
SEM knowledge	+ 3.2 points (*t*(5) = 3.63, *P* = 0.015)^2^	+ 2.5 points (*t*(20) = 4.07, *P* < 0.001)	+ 2.7 points (*t*(26) = 5.00, *P* < 0.001)
Self-efficacy	+ 4.8 points (*t*(5) = 3.24, *P* = 0.023)	+ 5.8 points (*t*(20) = 8.89, *P* < 0.001)	+ 5.5 points (*t*(26) = 9.28, *P* < 0.001)
Video modules completed by participants (percent completed)	6 (100%)	21 (100%)	27 (100%)

1 Note: Two participants completed the pre-course survey only.

2 Paired sample *t*-test: t(degrees of freedom) = t-value, *P*-value.

*SEM*, social emergency medicine.

### Feasibility and Acceptability

In the two cohorts combined, survey participants completed 100% of the video modules, while 27 of the 29 (93.1%) enrolled participants completed the post-course survey, exceeding our pre-established threshold for feasibility (Table 2). Twenty-five participants who completed the post-course survey (92.6%) felt the course content was important and that they would recommend the course to others, far exceeding our pre-established threshold for acceptability, while two participants (7.3%) agreed with these statements “somewhat.”

An overwhelming majority of participants (86%) felt that the course was organized in a manner that facilitated understanding the underlying concepts of SEM and felt the number of sessions (76%) and length of each session (79%) was “just right” (Table 3). Regarding the content of the modules, participants felt overall the modules effectively explained and illustrated the presented concepts (90%), contrasted the implications of various theories (90%), and adequately discussed current developments in the field (90%). See Tables 2 and 3 for complete quantitative results summary.

**Table 3.** Acceptability and organization responses regarding RISE-EM course (n = 27).

Acceptability questions			
Felt the course was important			
“yes”	6 (100%)	19 (90.5%)	25 (93%)
“somewhat”	0 (0%)	2 (9.5%)	2 (7%)
Would recommend the course to others			
“yes”	6 (100%)	19 (90.5%)	25 (93%)
“somewhat”	0 (0%)	2 (9.5%)	2 (7%)
Course organization questions			
Felt the course was organized in a helpful manner			
“yes”	5 (83%)	20 (95%)	25 (86%)
“somewhat”	1 (17%)	0 (0%)	1 (3%)
No response	0 (0%)	1 (5%)	1 (3%)
Felt the number of sessions was too many, just right, not enough			
“just right”	2 (33%)	20 (95%)	22 (76%)
“too many”	0 (0%)	1 (5%)	1 (3%)
“not enough”	4 (67%)	0 (0%)	4 (14%)
Felt the length of each session was too long, just right, too short			
“just right”	5 (83%)	18 (86%)	23 (79%)
“too long”	1 (17%)	3 (14%)	4 (13.8%)
Module content questions			
Modules effectively explained and illustrated the presented concepts			
“yes”	6 (100%)	20 (95%)	26 (90%)
“somewhat”	0 (0%)	1 (5%)	1 (3%)
Modules contrasted the implications of various theories			
“yes”	5 (83%)	19 (90%)	24 (83%)
“somewhat”	1 (17%)	2 (10%)	3 (10%)
Modules adequately discussed current developments in the field.			
“yes”	5 (83%)	21 (100%)	26 (90%)
“somewhat”	1 (17%)	0 (0%)	1 (3%)
Rate the level of instruction (just right, too basic)			
“just right”	6 (100%)	20 (95%)	26 (90%)
“too basic”	0 (0%)	1 (5%)	1 (3%)

### Qualitative Findings

For the five open-ended questions, 25 participants (five from Group 1 and 20 from Group 2 (86.2%) answered some or all of these questions (see Table 4). Regarding recommendations for course improvement, many responses suggested breaking content into different days or sessions to allow more time to process the content. Many participants also suggested that the instruction should include more examples of how to apply the content, including both case-based and action-focused examples. As one participant shared, it would be helpful to give “more specific examples. The ones provided were very helpful.” When asked about missing content, three participants again pointed to the benefit of including more examples, including “more concrete ways to incorporate SEM into my practice in a variety of settings.” Other, less common recommendations for improvement included a desire for a short quiz after each module and the suggestion to repeat key information more often across sessions.

**Table 4.** Top themes in qualitative responses, sorted by topic, with exemplar quotes.

Major themes, by question	Exemplar quote
Recommendations to improve, change (questions 1–2)	
Break content into different days, sessions	*“I would have liked to have done one at a time with a discussion between each.”*
More examples	*“More specific examples. The ones provided were very helpful.”*
Most useful content (question 3)	
Lecture 1 – introduction to SEM	*“Was the most generalizable for my ED and included the most hard facts that I was unaware of previously regarding the effects of homelessness.”*
Ways to take action as a clinician toward addressing SDoH	*“I think educating [clinicians] goes a long way, but in order to maximize the change in addressing SEM I think the rest of the ED staff should be included in these educational efforts.”*
Missing content (question 4)	
More real-life examples	*“More concrete ways to incorporate SEM into my practice in a variety of settings.”*
Nursing consideration and involvement	*“I think educating [clinicians] goes a long way, but in order to maximize the change in addressing SEM I think the rest of the ED staff should be included in these educational efforts.”*
Perspective change (question 5)	
Plans to implement a change in their clinical practice	*“Reinforced [SDoH] importance and has motivated me to consider SDoH in every patient and think more about how this is impacting their health and what my role is in addressing these in the ED.”*
The course reinforced the importance of SDoH	*“This course does a great job of raising awareness of the need for SEM, emphasizing the importance and feasibility of addressing it.”*

*SEM*, social emergency medicine; *ED*, emergency department; *SDoH*, social determinants of health.

In sharing the most helpful content, four participants appreciated the introduction to SDoH, which “was the most generalizable for my ED and included the hardest facts that I was unaware of previously regarding the effects of homelessness.” Three participants noted that other helpful content included ways to take action as a clinician toward addressing SDoH. When asked to describe how the course changed their views on social medicine, five participants reported plans to implement a change in their clinical practice, and five indicated that the course reinforced the importance of SDoH. For example, one participant stated that RISE-EM “reinforced [SDoH] importance and has motivated me to consider SDoH in every patient and think more about how this is impacting their health and what my role is in addressing these in the ED.”

## LIMITATIONS

The primary limitation of our research is the small sample size and self-selection of residents who chose to participate in this educational innovation. Participants with high interest in SEM material may have self-selected into the study, leading to higher ratings for acceptability. Given the importance we placed on ensuring that residents did not feel inappropriately compelled to participate, this was an anticipated result. Future testing of the intervention should incorporate larger and more diverse samples and may include testing in programs where completion of these modules is a mandatory component of training. Additionally, participants took the same test twice, which may have contributed to a practice effect that falsely elevated improvement. Future studies may incorporate a control group to compare improvement of those who receive the training as compared to those who complete the assessments only.

Another consideration is that Group 1 participants watched the videos in conference, in a single hour-long sitting, whereas the material was designed to be spaced out over four sessions. This format was chosen as it best met the needs and time available of the residency program at the time. Group 2 watched the videos asynchronously; so the time spent between video lectures was undefined. These differences in course delivery (conference vs home) and ability to space lectures out over time may have led to unmeasured differences in results based on training format.

## CONCLUSION

Leaders in emergency medicine and social medicine combined forces to create a new field of study, education, and interventions: social emergency medicine, the interaction between social factors and the emergency care system.^26^ Just as the field was in its early stages of development, COVID-19 struck, putting into the public eye social disparities and the growing burden on the emergency care system.^27^^–^^29^ The resulting wave of demand for addressing social medicine in the ED has trickled into resident education, as evidenced by the increased number of related fellowships and ACGME recommendations.^30^ Now, with growing awareness of the importance of addressing social determinants of health in EM, our video modules offer flexible, FOAMed resources to the program or clerkship director. Over 90% of participants felt the course content was important and would recommend the course to others. Furthermore, RISE-EM showed potential efficacy in improving SEM knowledge and growth in interest and self-efficacy in applying SEM competencies.

We identified a need for an easily implementable and educationally sound curriculum to improve knowledge of social determinants of health in EM training programs for both residents and medical students. We created a didactic video series with core content that can be integrated into existing EM training. The RISE-EM curriculum is a feasible, acceptable form of free open access medical education to assist in facilitating the development of SEM skills and self-efficacy for residents in their clinical practice. Residents demonstrated improved knowledge of SEM concepts and improved comfort in applying SEM to their practice. Given the participants in the study were recruited from two separate EM residencies, we feel that this curriculum is adaptable to other EM programs. In future studies we aim to include a larger sample size to allow for greater statistical power and more advanced statistical analysis, including assessing different delivery formats and evaluating differences in RISE-EM impact and outcomes based on various learner characteristics.

## Supplementary Information









