# Supplementary material for: RISE-EM: Resident Instruction in Social Emergency Medicine, a Cohort Study of a Novel Curriculum
Source: West J Emerg Med. 2024 Jun 11;25(4):593–601. doi: 10.5811/westjem.18103 (PMC11254142; doi:10.5811/westjem.18103)
Supplement: Supplementary file 1 [file wjem-25-593-s001.docx]

**All four RISE-EM video modules are compiled into a single video, viewed at the link below.**

[**https://drive.google.com/file/d/1K3lHoL4k7wb7t2cSKSfv9WmP0KMhV0YN/view?usp=sharing**](https://drive.google.com/file/d/1K3lHoL4k7wb7t2cSKSfv9WmP0KMhV0YN/view?usp=sharing)
