# Supplementary material for: RISE-EM: Resident Instruction in Social Emergency Medicine, a Cohort Study of a Novel Curriculum
Source: West J Emerg Med. 2024 Jun 11;25(4):593–601. doi: 10.5811/westjem.18103 (PMC11254142; doi:10.5811/westjem.18103)
Supplement: Supplementary file 2 [file wjem-25-593-s002.docx]

Dear resident,

We as a medical community are becoming increasingly aware of the large impact of social determinants of health on patient outcomes. This, combined with the severe health inequalities in our communities, means it is time to build social medicine training into our curriculums. In an effort to address this need, we are seeking to design a new social medicine curriculum specifically for emergency medicine residents.

As a step in addressing this change, we are inviting you to complete this research study, where you will view a 4-session curriculum aimed at empowering you to address the social determinants of health in your clinical practice, including homelessness, health disparities and treatment biases. Participation in the research study is entirely voluntary. Each lecture is approximately 20 minutes in length, and we would ask you to complete pre- and post-course surveys to test how well we’re doing with our project. Each survey will also take about 20 minutes to complete. This is the pilot run, and we’d love for you to see some early concepts that we’re working on, get your feedback, and to also let you influence our design.

The link below is to the pre-course survey. After you complete the pre-course survey, you will automatically be sent to a folder containing the videos. In conference on *** date, we will have a group discussion. You will be sent an email for the post-test and post-course survey after completion of the pre-course survey. Please complete this any time after watching the four videos.

[LINK TO CONSENT/PRE-COURSE SURVEY HERE]
