# Supplementary material for: RISE-EM: Resident Instruction in Social Emergency Medicine, a Cohort Study of a Novel Curriculum
Source: West J Emerg Med. 2024 Jun 11;25(4):593–601. doi: 10.5811/westjem.18103 (PMC11254142; doi:10.5811/westjem.18103)
Supplement: Supplementary file 3 [file wjem-25-593-s003.pptx]

## Slide 1
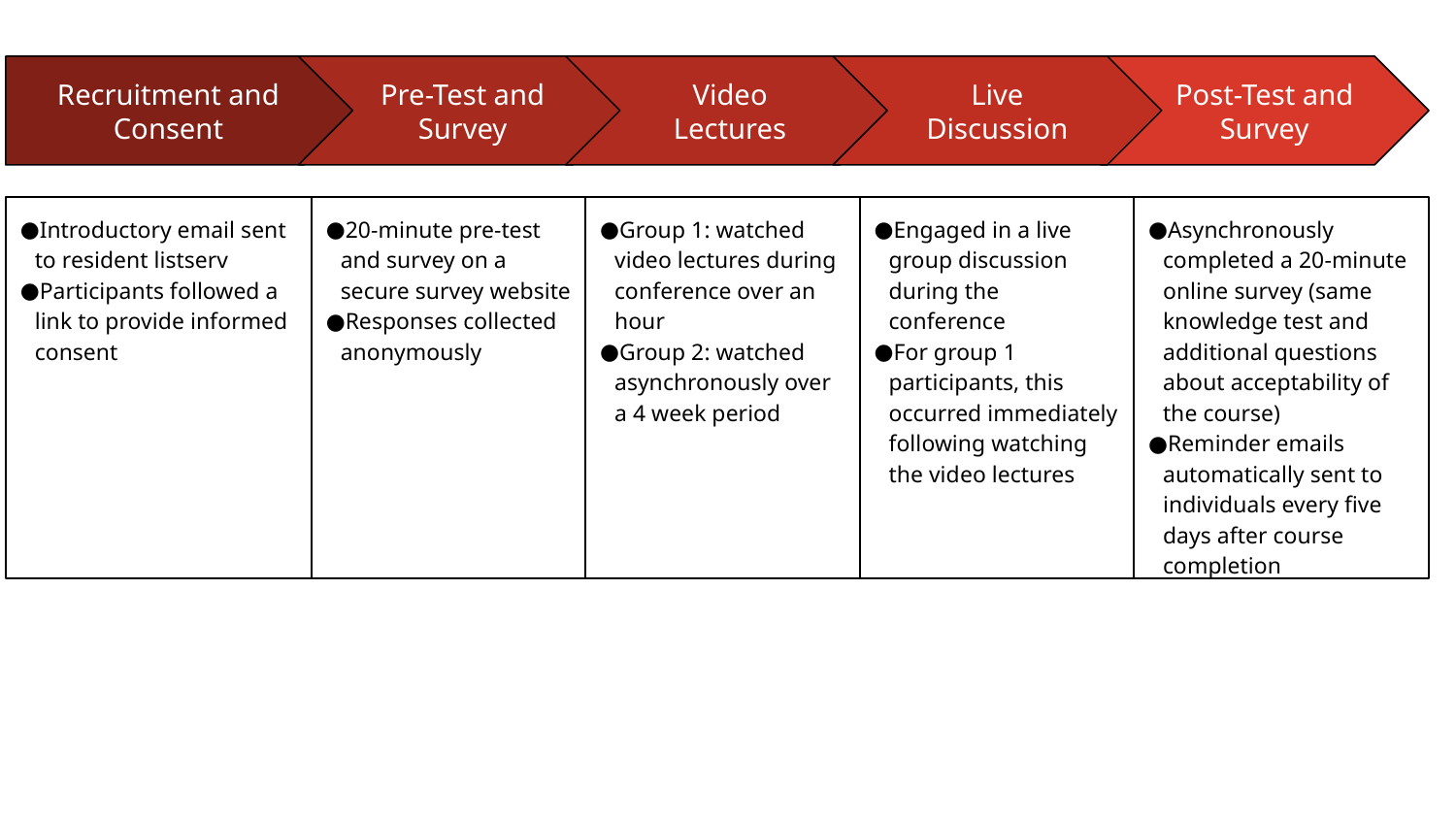

Pre-Test and Survey
20-minute pre-test and survey on a secure survey website
Responses collected anonymously
Video Lectures
Group 1: watched video lectures during conference over an hour
Group 2: watched asynchronously over a 4 week period
Live Discussion
Engaged in a live group discussion during the conference
For group 1 participants, this occurred immediately following watching the video lectures
Post-Test and Survey
Asynchronously completed a 20-minute online survey (same knowledge test and additional questions about acceptability of the course)
Reminder emails automatically sent to individuals every five days after course completion
Recruitment and Consent
Introductory email sent to resident listserv
Participants followed a link to provide informed consent
