# Supplementary material for: RISE-EM: Resident Instruction in Social Emergency Medicine, a Cohort Study of a Novel Curriculum
Source: West J Emerg Med. 2024 Jun 11;25(4):593–601. doi: 10.5811/westjem.18103 (PMC11254142; doi:10.5811/westjem.18103)
Supplement: Supplementary file 4 [file wjem-25-593-s004.docx]

**Pre-course Survey**

This information will be kept entirely anonymously in a secured database, separate from your test scores.

- I believe that social emergency medicine is important.
  - Yes
  - No
  - Somewhat
- I am interested in learning more about social emergency medicine.
  - Yes
  - No
  - Somewhat
- What is your age ______
- With which race/ethnicity do you identify? ___________
  - Prefer not to answer
- How Do you identify?
  - Male
  - Female
  - Other _____________________
- Have you had prior coursework or training in social medicine?
  - Yes
  - No
  - If so, describe:
- Have you had prior coursework or training in social emergency medicine?
  - Yes
  - No
  - If so, describe:
- What is your level of training?
  - PGY1
  - PGY2
  - PGY3
  - Faculty
  - Other _______________

*After the above has been completed, the next page of the survey will provide the link to the videos*

*redcap will send automated emails upon completion of the pre-course test with links to the post-course test and survey*
