# Supplementary material for: RISE-EM: Resident Instruction in Social Emergency Medicine, a Cohort Study of a Novel Curriculum
Source: West J Emerg Med. 2024 Jun 11;25(4):593–601. doi: 10.5811/westjem.18103 (PMC11254142; doi:10.5811/westjem.18103)
Supplement: Supplementary file 5 [file wjem-25-593-s005.docx]

We need to create a unique code for you that will not identify you personally. Please enter the last 4 digits of your phone number, followed by the first letter of your middle name.

______________

Thank you for participating in the pilot trial of our social emergency medicine (SEM) curriculum. In order to help us evaluate our course, please answer the following questions to the best of your knowledge.

1. Social determinants of health account for ___ of total health outcomes
   1. 40%
   2. 15%
   3. 25%
   4. 65%
2. An argument for addressing social determinants of health in the ED is
   1. Social and care workers are already built into the system
   2. Patients often have long wait times to see EM providers, which could be used more productively
   3. The ED is a strong social barometer of the community, and can be an important site for improving community health
   4. Primary care physicians are not trained to triage acute complaints like EM physicians
3. One of the quadruple aims of implementing SEM is
   1. Preventing provider burnout
   2. Decreasing unnecessary visits to the ED
   3. Increasing the resources available to patients upon discharge
   4. Improving provider efficiency and productivity
4. Why is addressing SDH useful in regards to ED overcrowding?
   1. Homeless patients are 8x more likely to visit the ED than their age and gender matched counterparts
   2. The relationships between SDH and ED recidivism is not well-established
   3. Overcrowding in the ED is largely due to a lack of PCP in many communities
   4. Interventions aimed at addressing SDH in the ED lead to 40% faster patient disposition, which reduces overcrowding
5. Which is/are true of SEM implementation?
   1. The primary focus of SEM implementation is on screening and referral for SDH in the ED
   2. Small actions, such as medical narratives, can be used to implement SEM
   3. SEM implementation requires substantive changes in ED [provider roles and workflow]
   4. Widespread implementation requires the support of physician leaders calling for policy change
   5. A and C
   6. B and D
   7. All of the above
6. Studies suggest that frequent fliers:
   1. Are unlikely to change their ED use pattern over time, often understand that their complaints aren’t very serious, and have typical mortality rates (compared to the general population)
   2. May change their ED pattern use over time, often believe their complaints are serious, and have high mortality rates (compared to the general population)
   3. Are unlikely to change their ED use pattern over time, often believe their complaints are serious, and have typical mortality rates (compared to the general population)
   4. May change their ED pattern use over time, often understand that their complaints aren’t that serious, and have high mortality rates (compared to the general population)

For questions 8-9, refer to the following vignette: A 65yo F PMH drug abuse and depression presents complaining of severe headache. Chart review notes she has presented to the ED several times in the last few months. Each time, the workup was negative for serious etiology, and the patient was discharged home.

1. As you ask questions, you note the patient has many relevant social determinants of health challenges. What is your next move?
   1. Get the social worker involved to ensure the social determinants of her health are addressed as you focus on the presenting complaint
   2. Create a differential diagnosis including the social determinants of health; order the SDH by severity to assess their impact on the patient’s health
   3. Look up community resources for the patient to connect with and provide her with the list upon discharge
   4. Consult with psychiatry to determine whether a psychiatric diagnosis and admission are appropriate for the patient
2. There are no red flags in the H&P, and you learn the patient recently became homeless. Which of the following might you include in your risk assessment when deciding patient disposition?
   1. Consider shelter hours prior to discharge
   2. Admission to the hospital may risk patient conflation of social and medical ills
   3. Inpatient evaluation of her social ills may be cheaper than decompensation due to unaddressed social determinants of health
   4. Discharging the patient reduces the risk of exposure to hospital bugs and deconditioning
   5. A and D
   6. A, C and D
   7. All of the above
3. What is nudge theory?
   1. A small change that makes it easier to voluntarily do the right thing
   2. Creating positive or negative reinforcements to encourage physicians to do the right thing
   3. Building an inexpensive, mandated behavior change into the system
   4. Incentivizing people to do the right thing with external rewards
4. Which of the following is a reliable proxy for estimating a patient's health literacy level?
   1. Age
   2. Gender
   3. Education level
   4. All of the above
   5. None of the above
5. Framing can occur via
   1. Reordering information in a patient presentation
   2. The use of quotes and descriptor words
   3. Physician interpretation of pain level
   4. Imaging results
   5. B,D
   6. A,B,C
   7. All of the above
6. Which of the following means of language interpretation has the potential to cause the most significant clinical errors?
   1. Ad hoc (i.e., family member, friend)
   2. Professional
   3. No interpreter
   4. Bilingual provider who has passed the translator test
   5. Bilingual employee that has taken HIPAA training
7. Which statement is true?
   1. Providers’ pain management practices are nearly identical for male and female patients in the ED
   2. Pain management is likely affected by bias, but research is inconclusive that bias causes treatment disparity
   3. Pain management disparities are worsened during ED overcrowding, indicating that biases are more pronounced when providers are under stress
   4. African Americans are statistically more likely to receive opioids in treatment of severe pain conditions, such as sickle cell disease
   5. A and C
   6. B and C
   7. All of the above
8. Effectively addressing social determinants of health means treating all patients equally, regardless of background
   1. True
   2. False
9. Which is true?
   1. The validity of screening tools for social determinants of health in the ED need additional evidence
   2. Screening for social determinants of health is a clear solution to addressing social ills in the ED
   3. You should only screen for social determinants of health in the ED if you have a strong referral for patients who screen positive
   4. You should only ask social determinants of health questions when using validated screening tools
10. Which set of statistics is **true** regarding NC?
    1. NC is near-average in the nation for rates of poverty; twice as many Black North Carolinians live below the poverty line as white
    2. NC has lower poverty than most U.S. states; a higher percentage of Black North Carolinians live below the poverty line than any other group
    3. NC has higher poverty than most U.S. states; a higher percentage of Latino North Carolinians live below the poverty line than any other group
    4. NC has higher poverty than most U.S. states; Black and Asian-American North Carolinians have a similar percentage below the poverty line
11. UNC’s Beacon program, which provides targeted support for interpersonal abuse and violence, is available to patients and employees only
    1. True
    2. False; patient’s families are included
12. Which of the following statements is **false** regarding expenditures for addressing social determinants of health in the ED?
    1. It is a resource-intensive and expensive, but worthwhile, investment
    2. Movement towards value-based care could encourage SEM to become incentivized
    3. Connecting patients to primary care doctors is a primary focus of resource expenditures in addressing SDH
    4. Financing social determinants of health treatment ultimately saves money
13. What are effective ways to address a patient's request for over the counter medications?
    1. Recommend websites with discounted drugs, such as GoodRx
    2. Explain local pharmacy policies that allow for reduced costs to residents and faculty
    3. Use medical narratives to bring public awareness to the effects of healthcare costs on patients
    4. Provide them with a prescription alternative
    5. A, B, and C
    6. All of the above

Written response:

The following questions are to gauge your knowledge of SEM beyond multiple choice questions. Please answer in a few sentences. Use the ranking to indicate your level of confidence in your ability to answer each question.

1. A patient previously seen in the ED for a benign complaint presents in worsened condition because they did not follow the discharge instructions. List 3 possible underlying causes.
   1. How confident did you feel answering this question?
      1. Very confident
      2. Somewhat confident
      3. Not confident
2. Give an example of an upstream policy change that affected the emergency medical system
   1. How confident are you about your answer?
      1. Very confident
      2. Somewhat confident
      3. Not confident
3. Describe a bedside factor that can affect patient health outcomes
   1. How confident are you about your answer?
      1. Very confident
      2. Somewhat confident
      3. Not confident
4. What is one tool for implementing SEM change in the ED?
   1. How confident are you about your answer?
      1. Very confident
      2. Somewhat confident
      3. Not confident
5. What is a way to implement SEM change outside the ED?
   1. How confident are you about your answer?
      1. Very confident
      2. Somewhat confident
      3. Not confident
6. How likely are you to engage in one of the practices or approaches discussed in this course?
   - 1. Very likely
     2. Somewhat likely
     3. Not sure
     4. Unlikely
     5. Very unlikely
7. Describe an innovative program or intervention that addresses social determinants of health in the ED.
   1. How confident are you about your answer?
      1. Very confident
      2. Somewhat confident
      3. Not confident
8. How confident are you about your ability to assess and address social determinants of health in your clinical encounters?
   1. Very confident
   2. Somewhat confident
   3. Not confident
   4. Why or why not? _____________
