# Supplementary material for: RISE-EM: Resident Instruction in Social Emergency Medicine, a Cohort Study of a Novel Curriculum
Source: West J Emerg Med. 2024 Jun 11;25(4):593–601. doi: 10.5811/westjem.18103 (PMC11254142; doi:10.5811/westjem.18103)
Supplement: Supplementary file 6 [file wjem-25-593-s006.docx]

We need to create a unique code for you that will not identify you personally. Please enter the last 4 digits of your phone number, followed by the first letter of your middle name.

---------------------------------

Thank you for volunteering your time to participate in this course. Social emergency medicine is a new, developing field and it will require constant reflection and flexible direction to move forward successfully. Therefore, meaningful input from you is essential for the improvement of this course and social emergency medicine as a whole. Your feedback is very important to us.

- Did you watch video 1?
  - Yes
  - No
- Did you watch video 2?
  - Yes
  - No
- Did you watch video 3?
  - Yes
  - No
- Did you watch video 4?
  - Yes
  - No
- I believe that what I learned in this course is important.
  - Yes
  - No
  - Somewhat
- Would you recommend this course to other emergency medicine providers? Why or why not?
  - Yes
  - No
  - Somewhat
  - Why or why not? _____________
- Will this course change your future practice? How so?
  - Yes
  - No
  - Somewhat
  - Why or why not? _____________
- The instructor was enthusiastic about the course material
  - Yes
  - No
  - Somewhat
- The course was organized in a manner that helped me understand the underlying concepts of social emergency medicine.
  - Yes
  - No
  - Somewhat
- These sessions were approximately 20 minutes long. Did you find the sessions were:
  - Too long
  - Too short
  - Just right
- We provided four sessions to present this content. Was the number of sessions:
  - Too many
  - Too few
  - Just right
- The instructor effectively explained and illustrated course concepts.
  - Yes
  - No
  - Somewhat
- The instructor contrasted the implications of various theories
  - Yes
  - No
  - Somewhat
- The instructor adequately discussed current developments in the field
  - Yes
  - No
  - Somewhat
- I felt the pre and post tests were a good reflection of the material presented
  - Yes
  - No
  - Somewhat
- How would you rate the level of instruction?
  - Too advanced
  - Too basic
  - Just right

Self-efficacy questions:

- Following my completion of this course, I feel confident in assessing and addressing social determinants of health in my clinical encounters
  - Yes
  - No
  - Somewhat
  - Why or why not? _____________

Qualitative Questions (~5):

- What specific recommendations do you have for improving this course?
- If you could change one thing about this course, what would it be?
- Which lecture content did you find most useful and why?
- What do you wish would have been covered more in the material? What was missing?
- In what way did this course change the way you view social medicine, if at all?
- Because of this course, I feel more confident in applying social emergency medicine to my future practice.
  - Yes
  - No
  - Somewhat
- This class has increased my interest in this field of study.
  - Yes
  - No
  - Somewhat
